# YaeB, Expressed in Response to the Acidic pH in Macrophages, Promotes Intracellular Replication and Virulence of *Salmonella* Typhimurium

**DOI:** 10.3390/ijms20184339

**Published:** 2019-09-04

**Authors:** Huan Zhang, Xiaorui Song, Peisheng Wang, Runxia Lv, Shuangshuang Ma, Lingyan Jiang

**Affiliations:** 1 TEDA Institute of Biological Sciences and Biotechnology, Nankai University, Tianjin 300457, China; 2 Tianjin Key Laboratory of Microbial Functional Genomics, Tianjin 300457, China; 3 The Key Laboratory of Molecular Microbiology and Technology, Ministry of Education, Nankai University, Tianjin 300457, China; 4 College of Life Sciences, Nankai University, Tianjin 300071, China

**Keywords:** *Salmonella* Typhimurium, *yaeB*, phosphate-specific transport system, acidic pH, growth in macrophages, H-NS, virulence

## Abstract

*Salmonella enterica* serovar Typhimurium is a facultative intracellular pathogen that infects humans and animals. Survival and growth in host macrophages represents a crucial step for *S*. Typhimurium virulence. Many genes that are essential for *S*. Typhimurium proliferation in macrophages and associated with virulence are highly expressed during the intracellular lifecycle. *yaeB*, which encodes an RNA methyltransferase, is also upregulated during *S*. Typhimurium growth in macrophages. However, the involvement of YaeB in *S*. Typhimurium pathogenicity is still unclear. In this study, we investigated the role of YaeB in *S*. Typhimurium virulence. Deletion of *yaeB* significantly impaired *S*. Typhimurium growth in macrophages and virulence in mice. The effect of *yaeB* on pathogenicity was related to its activation of *pstSCAB*, a phosphate (P_i_)-specific transport system that is verified here to be important for bacterial replication and virulence. Moreover, qRT-PCR data showed YaeB was induced by the acidic pH inside macrophages, and the acidic pH passed to YeaB through inhibiting global regulator histone-like nucleoid structuring (H-NS) which confirmed in this study can repress the expression of *yaeB*. Overall, these findings identified a new virulence regulatory network involving *yaeB* and provided valuable insights to the mechanisms through which acidic pH and low P_i_ regulate virulence.

## 1. Introduction

*Salmonella enterica* serovar Typhimurium (*S*. Typhimurium) is a significant food-borne pathogen that infects the intestinal epithelium via the *Salmonella* pathogenicity island (SPI)-1-encoded type 3 secretion system [1]. After passage through the intestinal epithelium, bacteria are incorporated mainly by macrophages [2]. The adaptation to host macrophages for growth is an essential step of *S*. Typhimurium pathogenesis, inducing systemic infections with fatal outcomes [3].

During intracellular growth, *S*. Typhimurium mostly resides within *Salmonella*-containing vacuoles (SCVs), which protects bacteria from killing by antimicrobial parasitic cell activities and facilitates bacterial survival and replication within macrophages [4]. SCV maturation is involved in acidification [5], preventing the generation of toxic free radicals, formation of *Salmonella*-induced filaments, resistance to antibacterial peptides and regulation of juxtanuclear positioning [6]. Typical characteristics of the mature SCV include high levels of K^+^; mildly acidic pH (5.5–4.9); low Mg^2+^, PO_4_^3−^, and Fe^2+^ levels [7,8]; and freely available oxygen [9].

Although phosphorous is scarce in SCVs, it is essential for bacterial growth. In bacteria, phosphate (P_i_) is a component of nucleotides and is involved in intracellular energy storage and nucleic acid and membrane phospholipid formation. Moreover, P_i_ also plays an important role in the post-translational modification system of bacterial two-component signal regulation [10]. For bacteria, the major source of P_i_ is the high uptake P_i_-specific transport (Pst) system [11]. However, it is unclear whether the Pst system influences *S*. Typhimurium intracellular growth.

*yaeB* encodes a tRNA methyltransferase that methylates t^6^A to form m^6^t^6^A in Thr tRNA in *Escherichia coli* (*E. coli*) [12]. The *yaeB* gene is induced 2.3-fold during *S*. Typhimurium growth in murine macrophages [13], implying the potential relevance of *yaeB* in the virulence of *S*. Typhimurium. However, the specific role of *yaeB* in mediating the virulence of this pathogen has not been specified. In this study, we evaluated the involvement of *yaeB* in *S*. Typhimurium virulence. The transcriptome data demonstrated that deletion of *yaeB* reduced the transcription of *pstSCAB*, which was confirmed in this study to be essential for *S*. Typhimurium virulence. Moreover, we found that YaeB was induced by the acidic pH within SCVs and directly regulated by histone-like nucleoid structuring (H-NS) protein.

## 2. Results

### 2.1. Deletion of YaeB Impaired S. Typhimurium Growth in Macrophages

**Figure 1:**
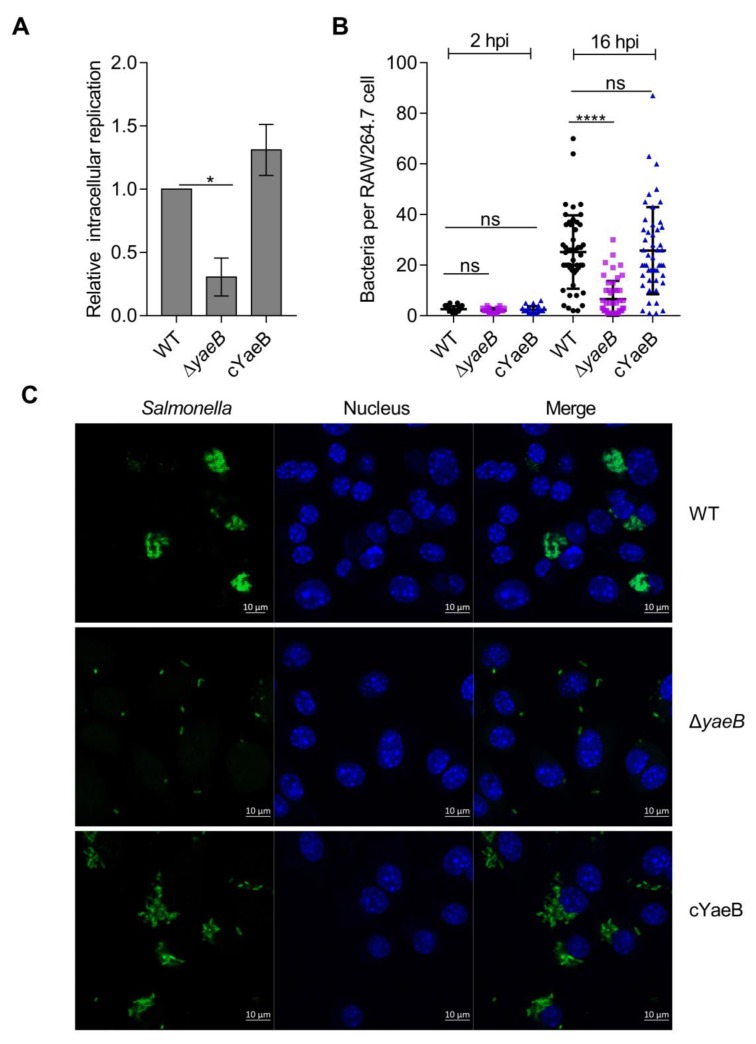
Deletion of *yaeB* reduced *S*. Typhimurium growth in RAW264.7 cells. (**A**) The intracellular growth of each strain was assessed according to the ratio of the number of intracellular bacterial cells at 16 hpi to the number of bacterial cells at 2 hpi. The replication abilities of the Δ*yaeB* and cYaeB strains were calculated relative to that of the WT strain. *P* values were determined by student’s *t*-tests. *P < 0.05. (**B**) Numbers of intracellular bacterial cells per RAW264.7 cell (at least 50 cells) were counted in random fields at 2 and 16 hpi. Bars show the mean numbers of bacterial cells contained in the infected RAW264.7 cells. *P* values were determined by student’s *t*-tests. ****P < 0.0001; ns, not significant. (**C**) Representative images of RAW264.7 cells infected by the WT, Δ*yaeB*, and cYaeB strains at 16hpi. Bacteria were labeled with anti-*Salmonella* lipopolysaccharide antibodies (green), and cell nuclei were counterstained with 4′,6-diamidino-2-phenylindole (blue).

To test whether *yaeB* influenced the growth of *S*. Typhimurium in macrophages, the growth rate of the *yaeB* mutant strain in RAW264.7 cells was examined. First, deletion of the *yaeB* gene did not affect bacterial growth in vitro (Figure S1). However, the growth rate of the *yaeB* mutant in RAW264.7 cells was reduced 3.0-fold (Figure 1A) compared with that in the wild-type (WT) strain; the growth rate of the complemented strain (cYaeB) was comparable to that of the WT strain (Figure 1A). Immunofluorescence microscopy examinations also confirmed the effect of *yaeB* deletion on *S*. Typhimurium intracellular proliferation. At 2 h post infection (hpi), each macrophage contained 1–4 WT bacterial cells or Δ*yaeB* (*yaeB* mutant strain) bacterial cells. However, at 16 hpi, an average of 27 WT bacterial cells were found in infected cells, whereas the average number of Δ*yaeB* bacterial cells was 7. In contrast, the average number of cYaeB bacterial cells was 26, which was comparable to that of the WT bacteria (Figure 1B,C). These results suggested that *yaeB* was critical for the growth of *S*. Typhimurium in macrophages.

### 2.2. Deletion of YaeB Impaired S. Typhimurium Virulence in Mice

**Figure 2:**
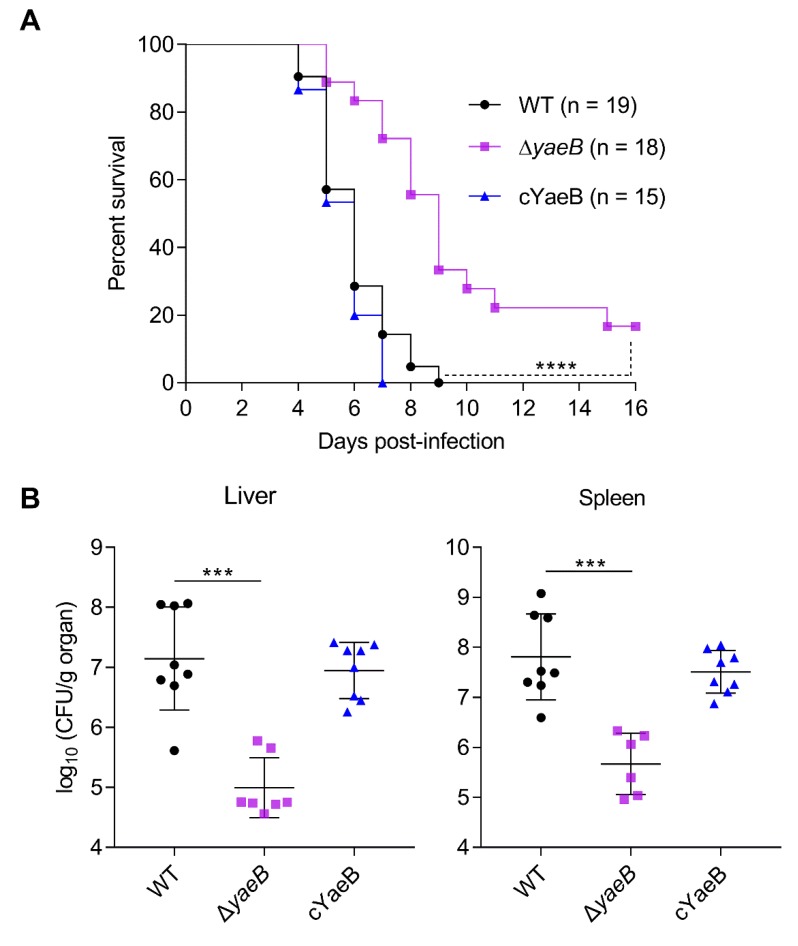
Deletion of *yaeB* attenuated *S*. Typhimurium virulence in mice. (**A**) To monitor survival, mice were i.p. injected with 2 × 10^4^ colony-forming units (CFU) of *S*. Typhimurium WT strain, *yaeB* mutant, or cYaeb strain. Log-rank curve comparison tests were used to calculate *P* values. ****P < 0.0001. (**B**) Bacterial counts recovered from the livers and spleens of mice infected by i.p. injection with 5 × 10^4^ CFU WT strain, *yaeB* mutant, or cYaeb strain at 3 dpi. Bars represent mean CFU of all mice, with significance determined by Mann–Whitney U tests. ***P < 0.001.

Because bacterial growth in macrophages is associated with systemic pathological processes, we further tested the growth fitness of the Δ*yaeB* mutant in a mouse model of infection. First, BALB/c mice were subjected to intraperitoneal (i.p.) injection with the WT, Δ*yaeB*, and cYaeB strains, and the survival of the infected mice was evaluated. The mice did not survive more than 9 days after i.p. inoculation with WT or cYaeB. Conversely, the survival rates of group inoculated with Δ*yaeB* were 20% at 16 days post infection (dpi; Figure 2A). In addition, we found that the bacterial loads were significantly lower in Δ*yaeB*-infected mice than in WT-infected mice (Figure 2B). Additionally, the colonization ability of cYaeB was similar to that of the WT strain (Figure 2B). These results revealed that deletion of *yaeB* seriously impaired *S*. Typhimurium virulence in mice.

### 2.3. Deletion of YaeB Reduced the Expression of the pstSCAB Gene

To identify the targets of *yaeB*, RNA-seq was performed to detect differences in global gene expression profiles between the WT strain and *yaeB* mutant strain. In total, 267 genes were differentially expressed, including 148 downregulated genes and 119 upregulated genes. Because the *yaeB* gene is required for the growth of *S.* Typhimurium in macrophage, we focus on the down-regulated genes in the *yaeB* mutant. These down-regulated genes are associated with biofilm synthesis (*leuO*) [14], glycerol triphosphate transport (*pgtC*, *pgtP*) [15,16], lysine transport (*cadB*, *cadA*), proline transport (*proV*, *proW*) [17], 3-phosphate glycerol transport (*ugpC*, *ugpE*, *ugpA*, *ugpB*, *glpT*) [18,19], inorganic phosphorus transport (*pstA*, *pstB*, *pstC*, *pstS*) [11,20], and bacterial specific antioxidant protein (*dps*) [21].

**Figure 3:**
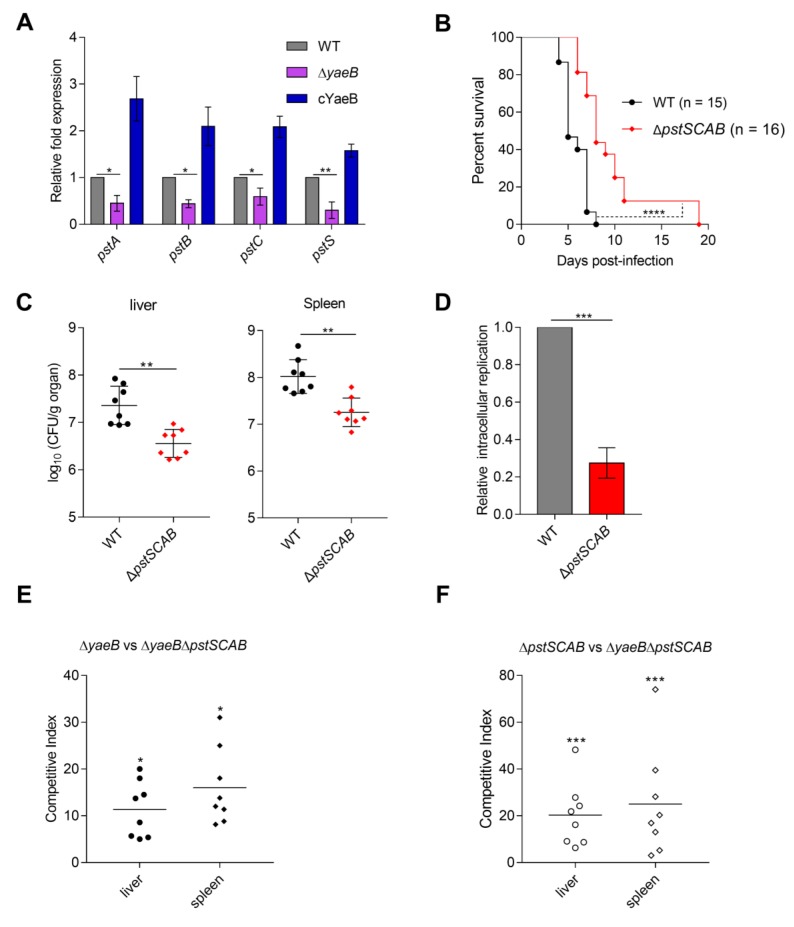
YaeB influenced virulence by activating *pstSCAB* genes. (**A**) Deletion of *yaeB* reduced the transcription of *pstSCAB* genes. Fold changes in gene transcription in the *yaeB* mutant or cYaeb strain relative to their expression in the WT strain are shown. Student’s *t*-tests were used to calculate *P* values. *P < 0.05; **P < 0.01. (**B**) Mice were infected by i.p. injection with 2 × 10^4^ CFU of the WT strain or *pstSCAB* mutant to monitor for survival. Log-rank curve comparison tests were used to calculate *P* values. ****P < 0.0001. (**C**) Bacterial counts recovered from the livers and spleens of mice injected i.p. with the WT or *pstSCAB* mutant strain at 3 dpi. Bars represent mean CFU of all mice, and significance was determined by Mann–Whitney U tests. **P < 0.01. (**D**) Growth of the WT strain or *pstSCAB* mutant strain in macrophages was determined by the ratio of the count of intracellular bacteria at 16 hpi to the count of bacteria at 2 hpi. The replication abilities of the mutant were calculated relative to that of the WT strain. Student’s *t*-tests were used to calculate *P* values. ***P < 0.001. (**E**,**F**) BALB/c mice were infected by i.p. injection with either a 1:1 mixture of the *yaeB* mutant and the *yaeB*/*pstSCAB* double mutant (**E**), or a 1:1 mixture of the *pstSCAB* mutant and the *yaeB*/*pstSCAB* double mutant (**F**) 2 × 10^4^ CFU in total, and sacrificed at 3 dpi for enumeration of bacterial burdens. CI values were determined in the liver and spleen. The data represent results combined from two independent experiments. The Mann–Whitney U test was used to calculate *P* values on raw CFU values, *P < 0.05; ***P < 0.001.

The *pstSCAB* gene in the Pst system, which is involved in high-affinity uptake of P_i_ [22] may associate with *S.* Typhimurium adaptation to the low phosphorus environment of SCVs. Previous reports indicated that Pst is involved in bacterial virulence [23,24]. Therefore, we posed the possibility that YaeB influences *S.* Typhimurium virulence by activating Pst system. Quantitative real-time PCR (qRT-PCR) analysis confirmed the downregulation of *pstA*, *pstB*, *pstC*, and *pstS* in the Δ*yaeB* relative to the WT strain (Figure 3A). Because of the low P_i_ conditions of SCVs, transport of P_i_ may play a significant role in the intracellular replication and virulence of *S*. Typhimurium. We confirmed that deletion of *pstSCAB* significantly impaired *S*. Typhimurium intracellular replication and virulence in mice (Figure 3B–D and Figure S2), implying that activation of Pst was important for virulence.

Next, to verify whether the attenuated virulence of the *yaeB* mutant depends on its regulation of Pst, we constructed a ∆*yaeB*∆*pstSCAB* double mutant strain and performed competitive infection assays between the ∆*yaeB*∆*pstSCAB* double mutant and each of the *yaeB* or *pstSCAB* single mutant. The competitive index (CI) values of the *yaeB* mutant versus the double mutant in livers (11.4) and spleens (16.1) (Figure 3E) were smaller than that of the *pstSCAB* mutant versus the double mutant (liver 20.5, spleen 25.1) (Figure 3F). The results suggested that the reduced virulence of the *yaeB* mutant was more remarkable than that of the *pstSCAB* mutant. Thus, *yaeB* contribution to bacterial virulence was partially dependent on *pstSCAB*.

### 2.4. YaeB was Directly Repressed by H-NS

**Figure 4:**
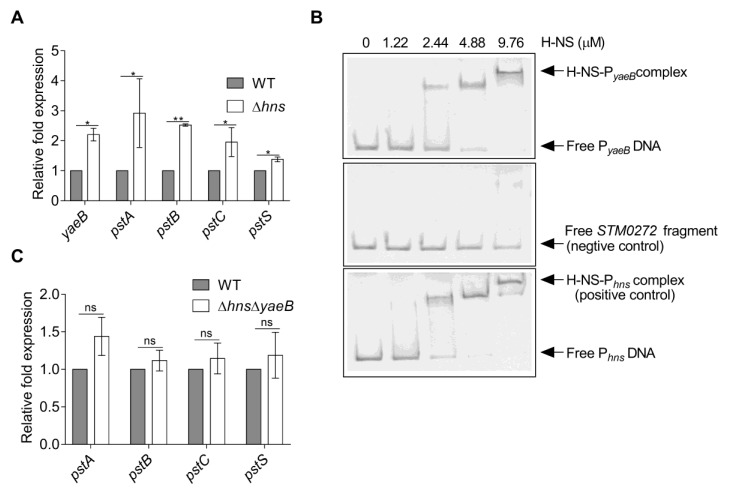
H-NS directly repressed *yaeB* gene expression. (**A**) Gene expression levels of *yaeB*, *pstA*, *pstB*, *pstC*, and *pstS* in WT and *hns* mutant strains were assessed by qRT-PCR. Relative fold changes in gene transcription in the *hns* mutant relative to their expression in the WT strain are presented. Student’s *t*-tests were used to calculate *P* values. *P < 0.05; **P < 0.01. (**B**) EMSAs of *yaeB* promoter DNA fragments with purified H-NS protein (0, 1.22, 2.44, 4.88, and 9.76 µM). The *hns* promoter was used as a positive control; the *STM0272* fragment was used as a negative control. (**C**) Gene expression levels of *pstA*, *pstB*, *pstC*, and *pstS* in WT and ∆*hns*∆*yaeB* strains were assessed by qRT-PCR. Relative fold changes in gene transcription in the ∆*hns*∆*yaeB* strain relative to their expression in the WT strain are presented. Student’s *t*-tests were used to calculate *P* values. ns, not significant.

Prediction of the upstream region of *yaeB* revealed putative H-NS (TAGGCTGA) binding sites (http://www.softberry.com/berry.phtml?topic=bprom&group=programs&subgroup=gfindb). Inactivation of *hns* in the WT strain resulted in a 2.2-fold increase in the expression of *yaeB,* revealing that H-NS repressed *yaeB* expression (Figure 4A). Electrophoretic mobility shift assays (EMSAs) revealed that H-NS bound specifically to the *hns* promoter (positive control) and *yaeB* promoter, but not to the *STM0272* fragment (negative control; Figure 4B) [25]. Taken together, our results revealed that H-NS directly repressed the expression of *yaeB* through binding to its promoter region.

Additional qRT-PCR experiments showed that the transcription levels of *pstA*, *pstB*, *pstC*, and *pstS* were also significantly increased in the *hns* mutant (Figure 4A). To investigate whether H-NS repression of Pst requires YaeB, we constructed the ∆*hns*∆*yaeB* double mutant strain and then tested the expression levels of *pstA*, *pstB*, *pstC*, and *pstS* in the double mutant and WT strain. The result showed that the expressions of *pstSCAB* genes were not significantly altered in the double mutant compared to the WT strain (Figure 4C), indicating that the deletion of *yaeB* in the *hns* mutant abolished the Pst induction, thus H-NS repression of Pst required YaeB.

### 2.5. YaeB Expression was Activated by an Acidic pH 

**Figure 5:**
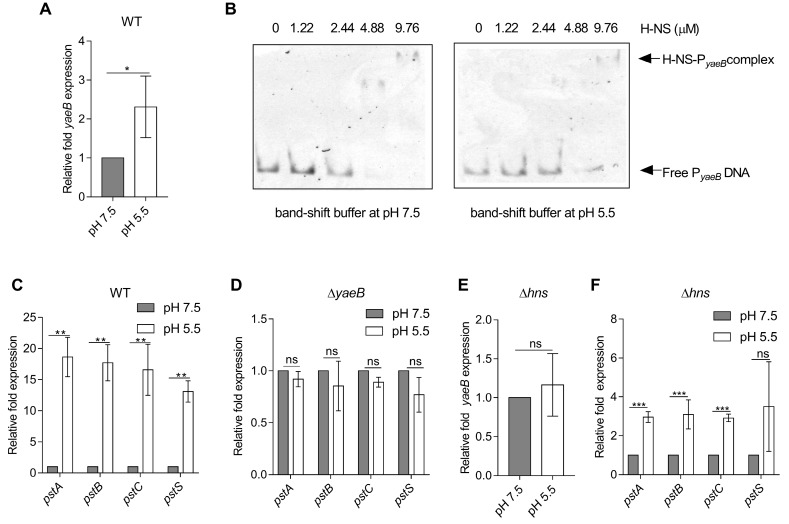
Acidic pH induced *yaeB* and *pstSCAB* gene expression. *S*. Typhimurium WT strain was cultured in N-minimal medium at pH 5.5 or 7.5 to the stationary phase. Fold changes in *yaeB* (**A**), *pstA*, *pstB*, *pstC*, and *pstS* (**C**) expression at pH 5.5 relative to that at pH 7.5 are presented. (**B**) EMSAs of *yaeB* promoter DNA fragments with purified H-NS protein (0, 1.22, 2.44, 4.88, and 9.76 µM) in band-shift buffer at pH 7.5 or pH 5.5. (**D**) Fold changes in *pstA*, *pstB*, *pstC*, and *pstS* expression at pH 5.5 relative to that at pH 7.5 after culture of the *yaeB* mutant strain in N-minimal medium to the stationary phase are presented. Fold changes in *yaeB* (**E**), *pstA*, *pstB*, *pstC*, and *pstS* (**F**) expression at pH 5.5 relative to that at pH 7.5 after culture of the *hns* mutant strain in N-minimal medium to the stationary phase are presented. Student’s *t*-tests were used to calculate *P* values. *P < 0.05; **P < 0.01; ***P < 0.001; ns, not significant.

We then investigated the environmental signals that induced *yaeB* expression. The regulatory activity of H-NS was previously reported to be influenced by acidic pH [26], which is a typical feature of the SCVs environment [27]. Through qRT-PCR analysis, we tested whether acidic pH influenced *yaeB* expression. Our results showed that the expression of *yaeB* was induced 2.3-fold when the WT strain was exposed to pH 5.5 compared with pH 7.5 (Figure 5A). We speculated that H-NS expression might be down-regulated in an acidic environment. However, the mRNA level of *hns* was not altered in response to acidic pH (Figure S3), consistent with the results of previous reports [28]. To investigate whether the acidic pH influences H-NS binding to the *yaeB* promoter, we performed the EMSAs experiment in band-shift buffer at pH 5.5 and pH 7.5. We observed that the affinity for H-NS protein and *yaeB* promoter DNA was weaker at pH 5.5 than that at pH 7.5 (Figure 5B), indicating the binding ability of H-NS to the *yaeB* promoter was weak at the acidic pH and thus the acid pH abolished the repression of *yaeB* by H-NS.

Consistent with the upregulation of *yaeB*, the expression of *pstSCAB* was highly induced (13.1–18.7-fold) by acidic pH (Figure 5C). These findings indicated that YaeB and Pst were upregulated in response to an acidic pH. To investigate whether the Pst induction in acidic pH is relying on YaeB, we compared the expression levels of *pstA*, *pstB*, *pstC*, and *pstS* in bacterial strains (WT and ∆*yaeB*), at pH 5.5 relative to that at pH 7.5. The result showed that the acidic pH induced *pstSCAB* gene expression in WT strain (Figure 5C), while the Pst induction by acidic pH was abolished in the *yaeB* mutant strain (Figure 5D), implying the *pstSCAB* induction in acidic pH required YaeB.

We further tested whether the acidic pH inducing *yaeB* is completely dependent on H-NS. We found that the expression of *yaeB* was not induced by acidic pH in an *hns* mutant strain (Figure 5E), indicating that acidic pH activated *yaeB* expression through H-NS. However, the expression of *pstSCAB* genes was also moderately induced (2.9–3.1-fold) (Figure 5F) by acidic pH in an *hns* mutant strain, indicating other regulators might also participate in the acidic pH-mediated Pst regulation system.

In addition, we showed that the deletion of *hns* and *yaeB* did not influence bacterial growth in N-minimal medium at both pH 7.5 or pH 5.5 (Figure S4), which excluded the possibility that their influences on p*stSCAB* gene expressions were due to a growth defect.

## 3. Discussion

**Figure 6:**
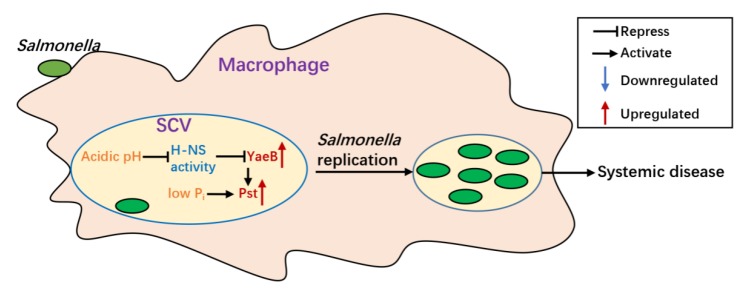
YaeB-mediated virulence model of *S*. Typhimurium. Once *S*. Typhimurium entered into macrophages, the acidic pH of SCVs relieved the inhibitory effects of H-NS on *yaeB*, and activation of YaeB resulted in upregulation of components of the Pst system, which promoted replication of *S*. Typhimurium within macrophages and accelerated *S*. Typhimurium spread and systemic infection.

In this study, we established that the N^6^-methyltransferase YaeB promoted the intracellular replication and systemic infection of *S*. Typhimurium. Activation of the Pst system was found to be responsible for the contribution of YaeB to intracellular replication and systemic infection. In addition*, yaeB* was directly inhibited by H-NS, and the inhibitory effect was abolished by the acidic pH inside SCVs. Thus, *yaeB* connected both the acidic pH and P_i_ regulatory system. We proposed a model for the *yaeB*-dependent *pstSCAB* regulatory pathway (Figure 6). Briefly, when *S*. Typhimurium is present within SCVs, the acidic pH relieves the inhibition of H-NS on *yaeB*, and activation of *yaeB* results in the upregulation of the Pst system, which enhances P_i_ acquisition and consequently increases the intracellular replication of *S*. Typhimurium.

The Pst system, a transport complex, is related to the activity of the Pho regulon [11]. When the P_i_ concentration is low, Pst can highly uptake P_i_, and under high P_i_ conditions, the expression of the Pho regulon is maintained at a basal level [29]. Previous studies have revealed that the Pst system is essential for the virulence of several pathogens. For example, deletion of the *pstSCAB* genes in an extra-intestinal pathogenic *E. coli* strain reduces virulence in a chicken infection model [23]. Mutants of *pstSCAB* in *Edwardsiella tarda* exhibit a reduced capacity to replicate within phagocytes and serum [24]. In addition, many virulence attributes, such as resistance to the bactericidal effects of serum, cationic antimicrobial peptides, and acidity, are affected by *pstSCAB* mutation [23]. In *S*. Typhimurium, *pstSCAB* genes have been reported to be moderately induced (~1.75–1.95-fold) during intracellular growth, and this system is highly (~5–8-fold) induced in response to Hank’s Balanced Salt Solution [30]. Moreover, recent transcriptome sequencing [13] and single-cell sequencing [31] studies have reported that *pstSCAB* gene expression is significantly upregulated during intracellular survival, thus suggesting that *S*. Typhimurium can be primed for host phosphate starvation and that *pstSCAB* may be essential for *S*. Typhimurim intracellular survival. In this study, we confirmed that deletion of the *pstSCAB* genes impaired *S*. Typhimurium replication in macrophages and virulence in vivo, demonstrating the involvement of *pstSCAB* in *S*. Typhimurium pathogenicity.

The exact mechanism through which YaeB activates *pstSCAB* genes in *S*. Typhimurium remains unclear. YaeB is known to methylate the N^6^-methyl group of m^6^t^6^A in tRNA Thr, which ensures efficient decoding and enhances the attenuation activity of the Thr operon, depending on stabilization of the anticodon by preventing fluctuations in the uracil base (U36) at the ribosomal A-site [12,32]. Therefore, it is possible that YaeB may affect tRNA methylation of an unknown regulatory protein, thereby altering the translation of *pstSCAB* genes in *S*. Typhimurium. Further studies are needed to define the direct targets of YaeB.

YaeB homologs are found in all members of the *Salmonella* genus, including *Salmonella enterica* and *Salmonella bongori*, and are also present in the commensal *E. coli* strain [12]. Notably, the YaeB protein from the nonpathogenic *E. coli* and pathogenic *Salmonella* exhibits high similarity (>89% amino acid identity; Figure S5), suggesting that *yaeB* may have been acquired before the divergence of *Salmonella* from *E.* *coli*. Thus, YaeB can not only promote the acquisition of P_i_ sources and promote colonization of nonpathogenic *E. coli* in the intestine but also contribute to the intracellular growth of *Salmonella*.

H-NS was originally found as a protein bound specifically to intrinsically curved DNA [33]. H-NS mainly negatively affects the transcription of numerous horizontal transfer genes that are generally associated with virulence [34]. H-NS is a global regulator that mediates the silencing of laterally acquired genes in bacteria. Whether the *hns* mutation is lethal in *Salmonella* is controversial in different published reports. Navarre et al. reported that *S.* Typhimurium *hns* mutant is nonviable unless additional mutations are present in either *rpoS* or *phoP* [35]. However, Ono et al. and Lucchini et al. indicated that the deletion of *hns* is not lethal in *S.* Typhimurium [36,37]. Our results also showed that the deletion of *hns* was not lethal in *S.* Typhimurium. The explanation for the different influence upon *hns* mutation in *Salmonella* requires further studies. Moreover, Navarre et al. reported that a ∆*hns*∆*rpoS* double mutant has reduced growth rates in rich media [35]. Here, by testing the growth rate of *hns* mutant generated in this study in vitro in LB medium and N-minimal medium, we found that *∆hns* grew as well as the WT strain in both culture conditions (Figure S6), indicating there are no growth defects caused by the *hns* mutation. It will be interesting to investigate the reason for the discrepancy in further studies.

Many H-NS regulatory genes are modulated by environmental signals, such as acidic pH, high osmolarity, and low oxygen [26]. However, the transcriptional level of *hns* has been shown to be unaffected by acidic pH, as was further confirmed in this study (Figure S3) [38], and H-NS can modulate the expression of *aniG* by altering operator topology under an acidic pH [28]. Our results indicated that H-NS induced *yaeB* expression by weakening its binding ability to *yaeB* promoter in response to a low pH. To exclude the possibility that the observed dysregulation of *yaeB* in the ∆*hns* strain is due to disruption of another mutation in passage, for example, *rpoS* and *phoP* as reported by Navarre [35]. We sequenced the *rpoS* and *phoP* genes of the *hns* mutant generated in this study and WT strain and found that mutations did not occur in *phoP* and *rpoS* loci (Figures S7 and S8), which supported our conclusion that the regulation of *yaeB* is mediated by H-NS.

In summary, our data demonstrated that the N^6^-methyltransferase YaeB contributed to *S*. Typhimurium virulence by influencing the expression of *pstSCAB* genes through unknown regulators. This regulatory mechanism was found to be critical for the pathogenicity of *S*. Typhimurium because lack of either *yaeB* or *pstSCAB* resulted in decreased virulence. The response to the mildly acidic pH resulted in activation of the system inside macrophages. Thus, our findings further deepened our understanding of how *S*. Typhimurium senses environmental signals to facilitate the overall adaptability of the pathogen.

## 4. Materials and Methods

### 4.1. Ethics Statement

All animal experiments were conducted in accordance with the criteria specified in the Guide for the Nursing and Use of Laboratory Animals. The experimental protocols were approved by the Institutional Animal Care Committee at Nankai University and Tianjin Institute of Pharmaceutical Research New Drug Evaluation Co. Ltd. (Tianjin, China; approval ID: IACUC 2015532198, validity period: 22 March 2015 to 22 March 2020).

### 4.2. Bacterial Strains and Plasmids

*S*. Typhimurium ATCC 14028s strain was used as a WT strain. Table 1 lists the bacterial strains and plasmids used in this study. The primers used in this study are shown in Table 2. Mutant strains were generated with the λ Red recombinase system [39] and then verified by sequencing. To generate the complementation strain of the *yaeB* mutant (cYaeB), the functional *yaeB* and its upstream promoter were amplified, digested with *Bam*HI and *Eco*RI restriction enzymes, and cloned into the pWSK129 plasmid [40] to obtain plasmid pYaeb. Then, pYaeb was transformed into the Δ*yaeB* strain to generate strain cYaeB. The pET-H-NS plasmid used to purify the H-NS-His_6_ protein was produced by cloning the *hns* gene sequence into the *Nde*I and *Xho*I sites downstream of the His-tag element in the pET28a plasmid. All the clones produced in this study were verified by DNA sequencing.

### 4.3. Bacterial Growth Culture and Cell Culture

Bacteria were generally grown in Luriae Bertani (LB) medium at 37 °C. However, strains containing the temperature-sensitive plasmid pKD46 were cultured at 30 ˚C. For RNA-seq and qRT-PCR analysis, bacteria were grown overnight in LB medium and inoculated 1:100 into fresh N-minimal medium (10 mM Tris-HCl pH 7.5, 5 mM KCl, 7.5 mM (NH_4_)_2_SO_4_, 0.5 mM K_2_SO_4_, 38 mM glycerol, 0.1% (*w/v*) casamino acids, 10 μM MgCl_2_, 1 mM KH_2_PO_4_) [41] (37 °C; 200 rpm) until reaching the stationary phase [42,43]. The working concentrations of antibiotics were 100 mg/L ampicillin (Ap), 50 mg/L kanamycin (Km), and 25 mg/L chloramphenicol (Cm). All bacterial strains were frozen at -80 °C with 15%–20% (*v/v*) glycerol.

RAW264.7 cells were purchased from Shanghai Institute of Biochemistry and Cell Biology of the Chinese Academy of Sciences (Shanghai, China) and grown in RPMI-1640 medium (Gibco, Thermo Fisher Scientific, Waltham, MA, USA) supplemented with 10% fetal bovine serum (Gibco; hereinafter referred to as complete medium) and incubated at 37 °C in an atmosphere containing 5% CO_2_/95% air. At 48 h before infection, cells were seeded into 24-well culture plates at a concentration of 5 × 10^4^ cells/well.

### 4.4. Macrophage Replication Assays

Different *S*. typhimurium strains were cultured in LB medium at 37 °C until reaching the stationary phase. Bacteria were precipitated in complete medium for 20 min and then added to a monolayer of RAW264.7 cells with a multiplicity of infection of 10. The culture plate was centrifuged at 800 × *g* for 5 min to synchronize the infection and incubated at 37 °C in an atmosphere containing 5% CO_2_/95% air for 45 min. The cells were washed twice with phosphate-buffered saline (PBS) to remove extracellular bacteria. The cells were then cultured in complete medium with high-concentration gentamicin (Gm) (100 μg/mL) for 2 h. Next, the cells were added to complete medium containing low-concentration Gm (10 μg/mL). At 2 and 16 hpi, the infected cells were washed three times in PBS and lysed for 5 min with 0.1% Triton X-100. Cell lysates were diluted continuously and plated on LB medium. CFU were counted after incubation at 37 °C overnight. Bacterial replication in cells was calculated by comparing the bacterial load at 16 h to that at 2 h.

### 4.5. Immunofluorescence Microscopy

RAW264.7 cells were plated on glass coverslips and infected as described above. At 2 and 16 hpi, cells were fixed with cold methyl alcohol (15 min at room temperature), treated with 3% H_2_O_2_ to inactivate endogenous peroxidase (10 min at room temperature), permeabilized with 0.1% Triton X-100 (20 min at room temperature), and blocked with 5% (*w/v*) bovine serum albumin in PBS (30 min at room temperature). Mouse anti-*Salmonella* lipopolysaccharide (Abcam, Cambridge, UK) was diluted 1:200 in PBS and applied (1 h at room temperature). Cells were washed three times with PBS and were then incubated with secondary goat anti-mouse IgG antibodies conjugated to fluorescein isothiocyanate (diluted 1:200 in PBS; Abcam) for 1 h to visualize bacteria. Cells were washed with PBS and incubated with 4′,6-diamidino-2-phenylindole (1 μg/mL; Invitrogen, Thermo Fisher Scientific, Waltham, MA, USA) for 2 min to visualize nuclear staining. After washing with PBS, cells were covered with 300 μL mounting medium and inspected for intracellular bacteria using an LSM 800 confocal laser scanning microscope (Zeiss, Oberkochen, Ostalbkreis, Baden-Württemberg, Germany).

### 4.6. Mouse Infections

Female BALB/c mice (ages 6–8 weeks) were purchased from Beijing Vital River Laboratory Animal Technology Co., Ltd. (Beijing, China). All mice were maintained in individually ventilated cage rack systems. Infection in mice was described previously [44]. In order to determine the survival rate, 0.1 mL of 0.9% (*w/v*) NaCl containing 2 × 10^4^ CFU overnight cultured bacteria was i.p. injected into mice. The status of mice was monitored daily, and the survival rate was recorded. In order to calculate the number of bacterial cells in liver and spleen, mice were infected by i.p. injection with 5 × 10^4^ CFU overnight cultured bacteria in 0.9% NaCl. At 3 dpi, the livers and spleens were harvested, homogenized in PBS, diluted, and plated on LB medium to determine the amount of CFU. The CI value was calculated as (CFU strain A recovered/CFU strain B recovered)/(CFU strain A inoculated/CFU strain B inoculated).

### 4.7. RNA-Seq

RNA was extracted using an RNeasy Mini Kit (Qiagen, Duesseldorf, Germany), followed by removal of residual genomic DNA with a Qiagen on-column RNase-Free DNase kit. Library construction was performed with a NEBNext Ultra Directional RNA Library Prep Kit for Illumina (NEB, lpswich, MA, USA). Sequencing was conducted on an Illumina Hiseq 1500 platform. Clean reads were obtained by removing reads containing adapter or poly-N sequences and low-quality reads from the raw data (in fastq format) using base calling. High quality reads were then mapped against the *S*. Typhimurium ATCC 14028s reference genome using Bowtie2-2.2.3 (https://sourceforge.net/projects/bowtie-bio/files/bowtie2/2.3.2/) [45]. The number of reads mapped to each gene was counted using HTSeq v0.6.1 (https://pypi.org/project/HTSeq/0.6.1/) Fragments per kilobase of transcript per million fragments mapped (FPKM) values were calculated to quantify the level of gene expression [46]. Differential expression analysis of the *yaeB* mutant compared to the WT strain was analyzed by the DESeq R package (1.18.0) (https://www.nuget.org/packages/PuppeteerSharp/1.18.0). Benjamini and Hochberg’s methods are used to adjust the generated *P* value to control the false discovery rate. Genes showing at least two-fold differences in FPKM values between two conditions were defined as differentially expressed. All sequence data were stored in the NCBI SRA database (registration number PRJNA545189).

### 4.8. qRT-PCR Analysis

Total RNA was purified using an RN43-EASY spin Plus Rapid Extraction Kit for Bacterial RNA (Aidlab, Beijing, China) according to the manufacturer’s instructions, and the quality of the RNA was determined using an ND-2000 spectrophotometer (NanoDrop Technologies, Montchanin, Delaware, USA). cDNA was synthesized using a PrimeScript RT reagent Kit with gDNA Eraser (TaKaRa, Beijing, China). qRT-PCR was performed using an ABI7500 system (Applied Biosystems, Foster City, CA, USA) with a total volume of 15 μL in a 96-well optical reaction plate (Applied Biosystems, Thermo Fisher Scientific, Waltham, MA, USA) containing 7.5 μL Universal SYBR Green PCR Master mix (Applied Biosystems), 1 μL cDNA, and 2 μL gene-specific primers. The primers used are shown in Table 2. The 16S rRNA gene was used as a reference, and fold changes in target gene expression were determined by the cycle threshold method (2^−∆∆*C*t^) [47].

### 4.9. EMSAs

The H-NS N-terminally tagged with 6× His fusion protein was expressed in *E. coli* BL21 containing pET-H-NS plasmid and purified from soluble extracts using a HiTrap Ni^2^^+^-chelating column, as previously described [48]. Protein concentrations were determined by the Bradford procedure, and the proteins were stored in aliquots at −80 °C. PCR fragments encompassing the regulatory regions of *hns* and *yaeB* and the *STM0272* fragment were amplified using the genomic DNA of the WT strain as a template. The fragments were then gel-purified, and 50 ng of the DNA fragments was incubated with purified protein (0–9.76 μM) in 20 μL of a solution containing band-shift buffer (20 mM Tris HCL, 1 mM ethylenediaminetetraacetic acid, 100 μg/mL bovine serum albumin, 1 mM dithiothreitol, 10% glycerol, and 80 mM NaCl [25]) at 25 °C for 30 min. Native 6% (*w/v*) polyacrylamide gels were used to separate the samples in 0.5 × Tris-borate-EDTA, and gels were then stained with Gel Green (Biotium, Fremont, CA, USA).

### 4.10. Statistical Analysis

GraphPad prism v7.0 software (https://www.graphpad.com/scientific-software/prism/) was used to analyze all data. Data are presented as means ± standard deviations from three independent experiments. Differences between two mean values were assessed by two-tailed student’s *t*-tests or Mann–Whitney U tests. Log-rank (Mantele–Cox) tests were used for survival comparisons. Results with *P* values of less than 0.05 were considered significant.

## Figures and Tables

**Table 1:** Bacterial strains and plasmids used in this study.

	Genotype or Description	Source
**S. Typhimurium strains**		
WT	Wild-type *S.* Typhimurium strain 14028s	Our lab
Δ*yaeB*	WT strain *yaeB*::Cm; Cm^R^	This study
Δ*pstSCAB*	WT strain *pstSCAB*::Km; Km^R^	This study
Δ*hns*	WT strain *hns*::Cm; Cm^R^	This study
Δ*yaeB*Δ*pstSCAB*	WT strain *yaeB*::Cm, *pstSCAB*::Km; Cm^R^_,_ Km^R^	This study
Δ*hns*Δ*yaeB*	WT strain *hns*::Cm; *yaeB*::Km; Cm^R^_,_ Km^R^	This study
cYaeB	Δy*aeB* harboring plasmid pYaeB; Cm^R^, Km^R^; complement strains	This study
BL21	For expressing protein	TransGen Biotech
**Plasmids**		
pKD4	For λRed recombination; Km^R^	Our lab
pKD3	For λRed recombination; CmR	Our lab
pWSK129	For generating complementation strains; Km^R^	Our lab
pKD46	For generating mutant strains with λ Red recombinase system under an arabinose-inducible promoter; Ap^R^	Our lab
pET28a	Expression vector; Km^R^	Our lab
pYaeB	pWSK129 carrying the WT *yaeB* gene; Km^R^	This study
pET-H-NS	pET28a carrying the WT *hns* gene; Km^R^	This study

**Table 2:** Primers used in this study.

Target Gene	Primer Sequence (5′–3′)		
Primers for construction of mutants^*^			
*yaeB*	F	ATGAGCAGCTTTCAGTTTGAACAAATCGGCGTTATTCGGTGTAGGCTGGAGCTGCTTCG	
	R	TTACCGTGGTTCCAGCGCAAACACTTCAAATCCCGAGTCATATGAATATCCTCCTTAG	
*pstSCAB*	F	ATGAAAGTTATGCGTACCACTGTCGCAACTGTTGTCGCGTGTAGGCTGGAGCTGCTTCG	
	R	TTAACCGTAACGACCGGTGATATAGTCTTCTGTTTGTTCATATGAATATCCTCCTTAG	
*hns*	F	ATGAGCGAAGCACTTAAAATTCTGAACAACATCCGTACGTGTAGGCTGGAGCTGCTTCG	
	R	TTATTCCTTGATCAGGAAATCTTCCAGTTGCTTACCTTCATATGAATATCCTCCTTAG	
***Primers for identification of mutants***			
*yaeB*	F	GCCAGGCTGCTATCGTCA	
	R	TGTTGTTACGGTTCCAGTT	
*pstSCAB*	F	CCTGGGCACGATTGTCTG	
	R	GCGGTGATAGCGTCAGAAA	
*hns*	F	CTCAACAAACCACCCCAATA	
	R	GGCTTGAAGAAGAAATGGGTA	
*rpoS*	F	ATAAGGAGTAACCGATGATTTG	
	R	CTACGCCCATAATGATACG	
*phoP*	F	TTTGCCCGTTTCATCGTA	
	R	TGCCGAAGGAATGAAACA	
***Primers for construction of clones and complemented strains***			
*yaeB*	F1		CGGGATCCACGCCAGCGAAAGACGGTAT
	R1	AATATTTCCTCAATGAATGT	
	F2	CAATATCTGGCAATTAGAACATTCATTGAGGAAATATTATGAGCAGCTTTCAGTTTGAA	
	R2	CCCAAGCTTTTACCGTGGTTCCAGCGCAAAC	
*hns-his_6_*	F	GGAATTCCATATGATGAGCGAAGCACTTAAAAT	
	R	CCGCTCGAGTTATTCCTTGATCAGGAAAT	
***qRT-PCR primers***			
*16S rRNA*	F	GAAAGCGTGGgGAGCAAAC	
	R	ACATGCTCCACCGCTTGTG	
*yaeB*	F	GCCTGGAAGCGTTCAGCCATT	
	R	CGAGCGACATGCCAATCGGATT	
*pstA*	F	GCTACGCTTGATATGCAGAACA	
	R	GCCATCGTCGCCATTGAGA	
*pstB*	F	ACGCCGTTCCCGATGTCCAT	
	R	ACAGACGCTGCTGCTGACCA	
*pstC*	F	GCTGATTGTGCTATTGATGT	
	R	GCGTCCCATTCCTTAGTC	
*pstS*	F	GCCAAATGGGCGGATACCTACC	
	R	TCACCGTCGGGAACTGGAACA	
*hns*	F	GCTGCTGCTGCTGAAGTGGAA	
	R	GCTGCGCGTTTAGCTTTGGTAC	
***Primers for EMSAs***			
P*_hns_*	F	CGACAGACGGTGAGTATC	
	R	TGCGTTCTTCCACTTCAG	
P*_yaeB_*	F	CAAAGACGCCGAGAATGTG	
	R	GCCAGATATTGAATCAGGAGC	
*STM0272*	F	CACCACGCTATTACTCACCACC	
	R	GCCGTGCTGTTCACTCATCC	

* Primers were designed to harbor extensions homologous to 38–40 bp (underlined) of the target gene; F, forward; R, reverse.

## Supplementary Materials

Supplementary Materials can be found at https://www.mdpi.com/1422-0067/20/18/4339/s1.

## Abbreviations

SCV	*Salmonella*-containing vacuole
qRT-PCR	quantitative real-time PCR
CFU	Colony-Forming Units
FPKM	fragments per kilobase of transcript per million fragments mapped
