# Supplementary material for: YaeB, Expressed in Response to the Acidic pH in Macrophages, Promotes Intracellular Replication and Virulence of Salmonella Typhimurium
Source: Int J Mol Sci. 2019 Sep 4;20(18):4339. doi: 10.3390/ijms20184339 (PMC6770890; doi:10.3390/ijms20184339)
Supplement: Supplementary file 1 [file ijms-20-04339-s001.pdf]

Supplementary Materials

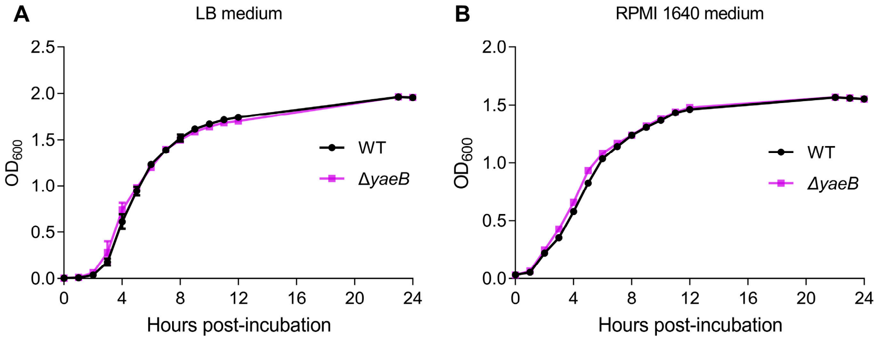

Figure S1. Deletion of *yaeB* did not influence *S.* Typhimurium growth in LB medium and RPMI-1640 medium. Overnight cultures of the WT strain and the *yaeB* mutant were subcultured 1:100 into fresh LB medium (A) or RPMI-1640 medium (B) and cultured for an additional 24 h at 37 °C with shaking at 200 rpm. The optical density at 600 nm (OD_{600}) of 1 ml aliquots of culture was measured regularly over this period.

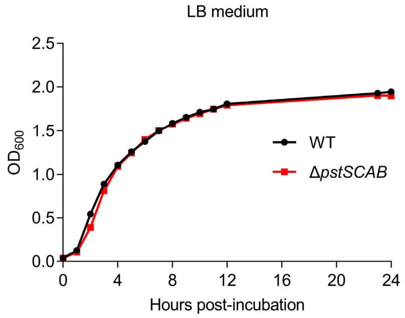

Figure S2. Deletion of *pst* genes did not influence *S.* Typhimurium growth in LB medium. Overnight cultures of the WT strain and the *pstSCAB* mutant were subcultured 1:100 into fresh LB medium and cultured for an additional 24 h at 37 °C with shaking at 200 rpm. The optical density at 600 nm (OD_{600}) of 1 ml aliquots of culture was measured regularly over this period.

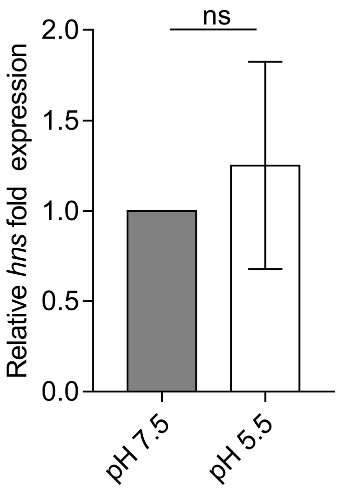

Figure S3. *hns* transcription was not induced by acidic pH. *S.* Typhimurium WT strain were cultured in N-minimal medium at pH 5.5 or pH7.5 to the stationary phase. Fold changes of *hns* expression at pH 5.5 relative to their expression at pH 7.5 are presented. ns, not significant.

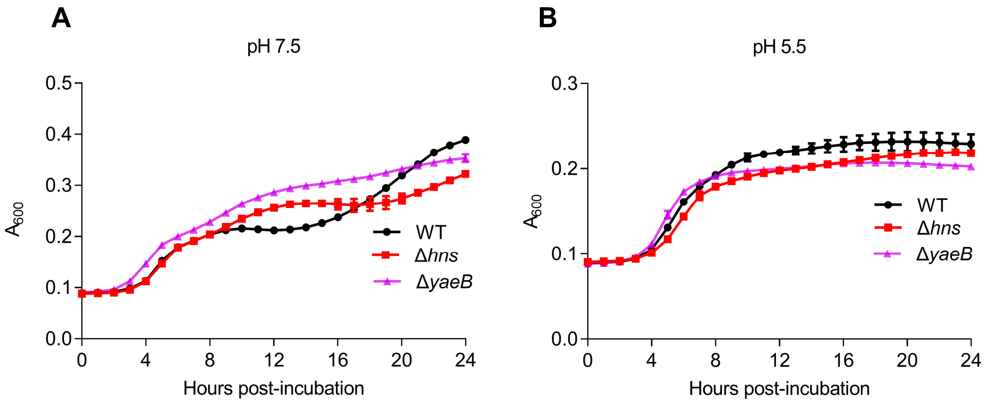

Figure 4. Deletion of *hns or yaeB* genes did not influence S. Typhimurium growth in N-minimal medium at pH 7.5 or pH 5.5. Overnight cultures of WT, Δ*hns* and Δ*yaeB* strains were subcultured 1:1000 into N-minimal medium at pH 7.5 (A) or at pH 5.5 (B) and cultured for an additional 24 h at 37 °C with shaking at 200 rpm. Measure the value of A_{600} every hour. The data represent results obtained from three independent experiments and are presented as means ± SD.

```
E.coli            MSSFQFEQIGVIRSPYKEKFAVPRQPGLVKSANGELHLIAPYNQADAVRGLEAFSHLWIL 60
S.Typhimurium     MSSFQFEQIGVIRSPYKEKFAVPRQPGLVKSACGELHLIAPYNQADAVRGLEAFSHLWVL 60
                  ******************************** *************************:*

E.coli            FVFHQTMEGGWRPTVRPPRLGGNARMGVFATRSTFRPNPIGMSLVELKEVVCHKDSVILK 120
S.Typhimurium     FVFHQTMDGGWRPTVRPPRLGGNARMGVFATRSTFRPNPIGMSLVALKGIECRKESVILK 120
                  *******:************************************* ** : *:*:*****

E.coli            LGSLDLVDGTPVVDIKPYLPFAESLPDASASYAQSAPAAEMAVSFTAEVEKQLLTLEKRY 180
S.Typhimurium     LGSLDLVDGTPVVDIKPYLPFAEALPDAAASYAQQAPIAEMPVSFTAEVAQQLTTLERRY 180
                  ***********************:****:*****.** *** ******* :** ***:**

E.coli            PQLTLFIREVLAQDPRPAYRKGEETGKTYAVWLHDFNVRWRVTDAGFEVFALEPR      235
S.Typhimurium     PQLRTFICDVLAQDPRPAYRKGEETGKTYAVWLHDFNVRWRVVNSGFEVFALEPR      235
                  ***  ** :*********************************.::**********
```

Figure 5. Sequence alignment of the amino acid sequences of YaeB of *E. coli* and *S*. Typhimurim using Clustal Omega (https://www.ebi.ac.uk/Tools/msa/clustalo/). Residues with * are strictly conserved and those with blank, . or, : indicate an amino acid substitution.

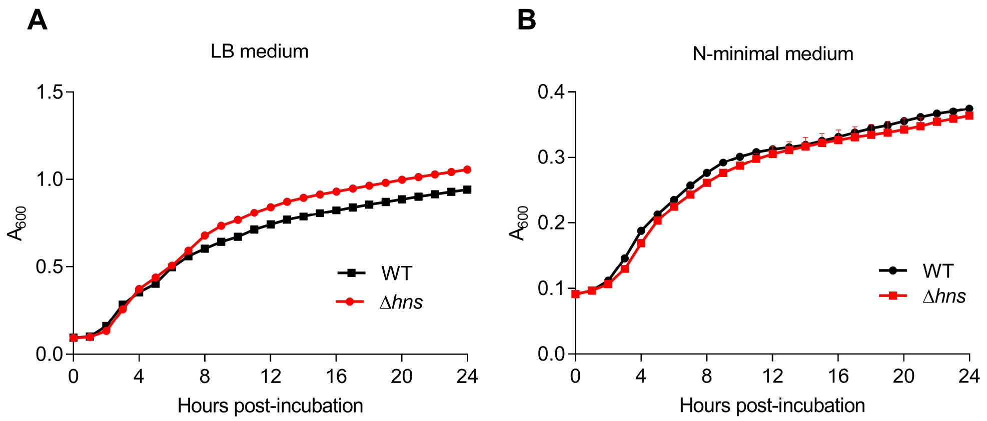

Figure S6. Deletion of *hns* did not influence S. Typhimurium growth in LB medium and N-minimal medium. Overnight cultures of the WT strain and the *hns* mutant were subcultured 1:100 into fresh LB medium (**A**) or N-minimal medium (**B**) and cultured for an additional 24 h at 37 °C with shaking at 200 rpm. Measure the value of A_{600} every hour. The data represent results obtained from three independent experiments and are presented as means ± SD.

```
CLUSTAL O(1.2.4) multiple sequence alignment

WT_rpos                 --------------TAGCACCGGCACCAGCTCTACACGCTTGCATTTTGAAATTCGTTAC    46
hns_mutant_rpos         ATCGCTACTATGGGTAGCACCGGCACCAGCTCTACACGCTTGCATTTTGAAATTCGTTAC    60
14028S_genome_rpos      ------------------------------------------------------------    0


WT_rpos                 AAGGGGAAATCCGTAAACCCGCTGCGTTATTTACCGCAGCGATAAAGCGGCGGAACCAGG    106
hns_mutant_rpos         AAGGGGAAATCCGTAAACCCGCTGCGTTATTTACCGCAGCGATAAAGCGGCGGAACCAGG    120
14028S_genome_rpos      ------------------------------------------------------------    0


WT_rpos                 CTTTGACTTGCTAGTTCCGTCAAGGGATCACGGGTAGGAGCCACCTTATGAGTCAGAATA    166
hns_mutant_rpos         CTTTGACTTGCTAGTTCCGTCAAGGGATCACGGGTAGGAGCCACCTTATGAGTCAGAATA    180
14028S_genome_rpos      -----------------------------------------------ATGAGTCAGAATA    13
                                                                       *************

WT_rpos                 CGCTGAAAGTTCATGATTTAAATGAAGACGCGGAATTTGATGAGAACGGAGTAGAGGCTT    226
hns_mutant_rpos         CGCTGAAAGTTCATGATTTAAATGAAGACGCGGAATTTGATGAGAACGGAGTAGAGGCTT    240
14028S_genome_rpos      CGCTGAAAGTTCATGATTTAAATGAAGACGCGGAATTTGATGAGAACGGAGTAGAGGCTT    73
                        ************************************************************

WT_rpos                 TTGACGAAAAAGCCTTGAGTGAAGAGGAACCCAGTGATAACGACCTGGCTGAAGAAGAGC    286
hns_mutant_rpos         TTGACGAAAAAGCCTTGAGTGAAGAGGAACCCAGTGATAACGACCTGGCTGAAGAAGAGC    300
14028S_genome_rpos      TTGACGAAAAAGCCTTGAGTGAAGAGGAACCCAGTGATAACGACCTGGCTGAAGAAGAGC    133
                        ************************************************************

WT_rpos                 TGTTATCGCAAGGGGCCACACAGCGTGTGTTGGACGCGACTCAGCTTTACCTTGGTGAGA    346
hns_mutant_rpos         TGTTATCGCAAGGGGCCACACAGCGTGTGTTGGACGCGACTCAGCTTTACCTTGGTGAGA    360
14028S_genome_rpos      TGTTATCGCAAGGGGCCACACAGCGTGTGTTGGACGCGACTCAGCTTTACCTTGGTGAGA    193
                        ************************************************************

WT_rpos                 TTGGGTATTCACCACTGTTAACAGCCGAAGAAGAAGTCTATTTTGCGCGTCGCGCACTGC    406
hns_mutant_rpos         TTGGGTATTCACCACTGTTAACAGCCGAAGAAGAAGTCTATTTTGCGCGTCGCGCACTGC    420
14028S_genome_rpos      TTGGGTATTCACCACTGTTAACAGCCGAAGAAGAAGTCTATTTTGCGCGTCGCGCACTGC    253
                        ************************************************************

WT_rpos                 GTGGAGATGTCGCTTCTCGCCGTCGCATGATTGAGAGTAACCTGCGTCTGGTGGTAAAAA    466
hns_mutant_rpos         GTGGAGATGTCGCTTCTCGCCGTCGCATGATTGAGAGTAACCTGCGTCTGGTGGTAAAAA    480
14028S_genome_rpos      GTGGAGATGTCGCTTCTCGCCGTCGCATGATTGAGAGTAACCTGCGTCTGGTGGTAAAAA    313
                        ************************************************************

WT_rpos                 TTGCCCGCCGTTATGGCAATCGTGGACTGGCGTTGCTGGACCTGATTGAAGAGGGCAACC    526
hns_mutant_rpos         TTGCCCGCCGTTATGGCAATCGTGGACTGGCGTTGCTGGACCTGATTGAAGAGGGCAACC    540
14028S_genome_rpos      TTGCCCGCCGTTATGGCAATCGTGGACTGGCGTTGCTGGACCTGATTGAAGAGGGCAACC    373
                        ************************************************************

WT_rpos                 TGGGGCTTATCCGTGCAGTCGAGAAGTTTGACCCGGAACGCGGGTTCCGCTTCTCAACAT    586
hns_mutant_rpos         TGGGGCTTATCCGTGCAGTCGAGAAGTTTGACCCGGAACGCGGGTTCCGCTTCTCAACAT    600
14028S_genome_rpos      TGGGGCTTATCCGTGCAGTCGAGAAGTTTGACCCGGAACGCGGGTTCCGCTTCTCAACAT    433
                        ************************************************************

WT_rpos                 ACGCAACCTGGTGGATTCGCCAGACAATCGAACGGGCGATCATGAACCAAACCCGTACGA    646
hns_mutant_rpos         ACGCAACCTGGTGGATTCGCCAGACAATCGAACGGGCGATCATGAACCAAACCCGTACGA    660
14028S_genome_rpos      ACGCAACCTGGTGGATTCGCCAGACAATCGAACGGGCGATCATGAACCAAACCCGTACGA    493
                        ************************************************************

WT_rpos                 TTCGCTTGCCGATTCACATTGTTAAAGAGCTGAACGTATACCTGCGCACCGCACGTGAGT    706
hns_mutant_rpos         TTCGCTTGCCGATTCACATTGTTAAAGAGCTGAACGTATACCTGCGCACCGCACGTGAGT    720
14028S_genome_rpos      TTCGCTTGCCGATTCACATTGTTAAAGAGCTGAACGTATACCTGCGCACCGCACGTGAGT    553
                        ************************************************************

WT_rpos                 TGTCGCATAAACTGGACCACGAACCGAGTGCGGAAGAAATTGCAGAGCAACTGGATAAAC    766
hns_mutant_rpos         TGTCGCATAAACTGGACCACGAACCGAGTGCGGAAGAAATTGCAGAGCAACTGGATAAAC    780
14028S_genome_rpos      TGTCGCATAAACTGGACCACGAACCGAGTGCGGAAGAAATTGCAGAGCAACTGGATAAAC    613
                        ************************************************************

WT_rpos                 CGGTTGATGACGTCAGCCGTATGCTTCGTCTCAACGAGCGCATTACCTCGGTAGACACCC    826
hns_mutant_rpos         CGGTTGATGACGTCAGCCGTATGCTTCGTCTCAACGAGCGCATTACCTCGGTAGACACCC    840
14028S_genome_rpos      CGGTTGATGACGTCAGCCGTATGCTTCGTCTCAACGAGCGCATTACCTCGGTAGACACCC    673
                        ************************************************************

WT_rpos                 CGCTGGGCGGTGATTCCGAAAAAGCGTTGCTGGACATCCTGGCCGATGAAAAAGAGAACG    886
hns_mutant_rpos         CGCTGGGCGGTGATTCCGAAAAAGCGTTGCTGGACATCCTGGCCGATGAAAAAGAGAACG    900
14028S_genome_rpos      CGCTGGGCGGTGATTCCGAAAAAGCGTTGCTGGACATCCTGGCCGATGAAAAAGAGAACG    733
                        ************************************************************

WT_rpos                 GTCCGGAAGACACCACGCAAGATGACGATATGAAACAGAGCATCGTCAAATGGTTGTTCG    946
hns_mutant_rpos         GTCCGGAAGACACCACGCAAGATGACGATATGAAACAGAGCATCGTCAAATGGTTGTTCG    960
14028S_genome_rpos      GTCCGGAAGACACCACGCAAGATGACGATATGAAACAGAGCATCGTCAAATGGTTGTTCG    793
                        ************************************************************

WT_rpos                 AACTGAACGCCAAACAGCGTGAAGTGCTGGCGCGCCGTTTCGGTCTGCTGGGATATGAAG    1006
hns_mutant_rpos         AACTGAACGCCAAACAGCGTGAAGTGCTGGCGCGCCGTTTCGGTCTGCTGGGATATGAAG    1020
14028S_genome_rpos      AACTGAACGCCAAACAGCGTGAAGTGCTGGCGCGCCGTTTCGGTCTGCTGGGATATGAAG    853
                        ************************************************************

WT_rpos                 CTGCGACACTGGAAGATGTAGGCCGTGAAATCGGTCTTACGCGTGAACGTGTTCGTCAGA    1066
hns_mutant_rpos         CTGCGACACTGGAAGATGTAGGCCGTGAAATCGGTCTTACGCGTGAACGTGTTCGTCAGA    1080
14028S_genome_rpos      CTGCGACACTGGAAGATGTAGGCCGTGAAATCGGTCTTACGCGTGAACGTGTTCGTCAGA    913
                        ************************************************************

WT_rpos                 TTCAGGTTGAAGGCCTGCGCCGTCTGCGCGAAATTCTGCAGACGCAGGGGCTGAATATCG    1126
hns_mutant_rpos         TTCAGGTTGAAGGCCTGCGCCGTCTGCGCGAAATTCTGCAGACGCAGGGGCTGAATATCG    1140
14028S_genome_rpos      TTCAGGTTGAAGGCCTGCGCCGTCTGCGCGAAATTCTGCAGACGCAGGGGCTGAATATCG    973
                        ************************************************************

WT_rpos                 AAGCGCTGTTCCGCGAGTAAGTACCCTTGTCAAAAAAAGGCCAGTCTGTCGACTGGCCTT    1186
hns_mutant_rpos         AAGCGCTGTTCCGCGAGTAAGTACCCTTGTCAAAAAAAGGCCAGTCTGTCGACTGGCCTT    1200
14028S_genome_rpos      AAGCGCTGTTCCGCGAGTAA----------------------------------------    993
                        ********************

WT_rpos                 TTTTTTACCGTTTGTCTTCTGCCAGCAGCGGCGGGATACTCGGCACCATTGGCGCGTCAT    1246
hns_mutant_rpos         TTTTTTACCGTTTGTCTTCTGCCAGCAGCGGCGGGATACTCGGCACCATTGGCGCGTCAT    1260
14028S_genome_rpos      ------------------------------------------------------------    993


WT_rpos                 CAATATCTTTTTGCGTCATGCGAAACGCTTGTGGATAGTGTTCGCGGCTGGTACGGCGTA    1306
hns_mutant_rpos         CAATATCTTTTTGCGTCATGCGAAACGCTTGTGGATAGTGTTCGCGGCTGGTACGGCGTA    1320
14028S_genome_rpos      ------------------------------------------------------------    993


WT_rpos                 ACGGTTCGGTATCGCGCCAGGTATACAGACAATGCTGACACTGATATACCGTCCAGACTC    1366
hns_mutant_rpos         ACGGTTCGGTATCGCGCCAGGTATACAGACAATGCTGACACTGATATACCGTCCAGACTC    1380
14028S_genome_rpos      ------------------------------------------------------------    993


WT_rpos                 CTTTTACCGGCGAAGTCGCC    1386
hns_mutant_rpos         CTTTTACCGGCGAAGTCGCC    1400
14028S_genome_rpos      --------------------    993
```

Figure S7. Mutation was not occurred in *rpoS* loci in the *hns* mutant strain**.** Sequence alignment of *rpoS* gene of the hns mutant, WT strain and *S*. Typhimurium ATCC 14028s strain genome using Clustal Omega (https://www.ebi.ac.uk/Tools/msa/clustalo/). Bases with * are strictly conserved.

```
CLUSTAL O(1.2.4) multiple sequence alignment

WT_phop             GCCATGACGCCGGCAAATTATATCGGTCGCGCTGTGACTCTGGTCGACGAACTTAAATAA   60
hns_mutant_phop     -------------------ATATCGGTCGCGCTGTGACTCTGGTCGACGAACTTAAATAA   41
14028S_genome_phop  ------------------------------------------------------------   0

WT_phop             TGCCTGCCTCACCCTCTTTTCTTCAGAAAGAGGGTGACTATTTGTCTGGTTTATTAACTG   120
hns_mutant_phop     TGCCTGCCTCACCCTCTTTTCTTCAGAAAGAGGGTGACTATTTGTCTGGTTTATTAACTG   101
14028S_genome_phop  ------------------------------------------------------------   0

WT_phop             TTTATCCCCAAAGCACCATAATCAACGCTAGACTGTTCTTATTGTTAACACAAGGGAGAA   180
hns_mutant_phop     TTTATCCCCAAAGCACCATAATCAACGCTAGACTGTTCTTATTGTTAACACAAGGGAGAA   161
14028S_genome_phop  ------------------------------------------------------------   0

WT_phop             GAGATGATGCGCGTACTGGTTGTAGAGGATAATGCATTATTACGCCACCACCTGAAGGTT   240
hns_mutant_phop     GAGATGATGCGCGTACTGGTTGTAGAGGATAATGCATTATTACGCCACCACCTGAAGGTT   221
14028S_genome_phop  ---ATGATGCGCGTACTGGTTGTAGAGGATAATGCATTATTACGCCACCACCTGAAGGTT   57
                       *********************************************************

WT_phop             CAGCTCCAGGATTCAGGTCACCAGGTCGATGCCGCAGAAGATGCCAGGGAAGCTGATTAC   300
hns_mutant_phop     CAGCTCCAGGATTCAGGTCACCAGGTCGATGCCGCAGAAGATGCCAGGGAAGCTGATTAC   281
14028S_genome_phop  CAGCTCCAGGATTCAGGTCACCAGGTCGATGCCGCAGAAGATGCCAGGGAAGCTGATTAC   117
                    ************************************************************

WT_phop             TACCTTAATGAACACCTTCCGGATATCGCTATTGTCGATTTAGGTCTGCCGGATGAAGAC   360
hns_mutant_phop     TACCTTAATGAACACCTTCCGGATATCGCTATTGTCGATTTAGGTCTGCCGGATGAAGAC   341
14028S_genome_phop  TACCTTAATGAACACCTTCCGGATATCGCTATTGTCGATTTAGGTCTGCCGGATGAAGAC   177
                    ************************************************************

WT_phop             GGCCTTTCCTTAATACGCCGCTGGCGCAGCAGTGATGTTTCACTGCCGGTTCTGGTGTTA   420
hns_mutant_phop     GGCCTTTCCTTAATACGCCGCTGGCGCAGCAGTGATGTTTCACTGCCGGTTCTGGTGTTA   401
14028S_genome_phop  GGCCTTTCCTTAATACGCCGCTGGCGCAGCAGTGATGTTTCACTGCCGGTTCTGGTGTTA   237
                    ************************************************************

WT_phop             ACCGCGCGCGAAGGCTGGCAGGATAAAGTCGAGGTTCTCAGCTCCGGGGCCGATGACTAC   480
hns_mutant_phop     ACCGCGCGCGAAGGCTGGCAGGATAAAGTCGAGGTTCTCAGCTCCGGGGCCGATGACTAC   461
14028S_genome_phop  ACCGCGCGCGAAGGCTGGCAGGATAAAGTCGAGGTTCTCAGCTCCGGGGCCGATGACTAC   297
                    ************************************************************

WT_phop             GTGACGAAGCCATTCCACATCGAAGAGGTAATGGCGCGTATGCAGGCGTTAATGCGCCGT   540
hns_mutant_phop     GTGACGAAGCCATTCCACATCGAAGAGGTAATGGCGCGTATGCAGGCGTTAATGCGCCGT   521
14028S_genome_phop  GTGACGAAGCCATTCCACATCGAAGAGGTAATGGCGCGTATGCAGGCGTTAATGCGCCGT   357
                    ************************************************************

WT_phop             AATAGCGGTCTGGCCTCCCAGGTGATCAACATCCCGCCGTTCCAGGTGGATCTCTCACGC   600
hns_mutant_phop     AATAGCGGTCTGGCCTCCCAGGTGATCAACATCCCGCCGTTCCAGGTGGATCTCTCACGC   581
14028S_genome_phop  AATAGCGGTCTGGCCTCCCAGGTGATCAACATCCCGCCGTTCCAGGTGGATCTCTCACGC   417
                    ************************************************************

WT_phop             CGGGAATTATCCGTCAATGAAGAGGTCATCAAACTCACGGCGTTCGAATACACCATTATG   660
hns_mutant_phop     CGGGAATTATCCGTCAATGAAGAGGTCATCAAACTCACGGCGTTCGAATACACCATTATG   641
14028S_genome_phop  CGGGAATTATCCGTCAATGAAGAGGTCATCAAACTCACGGCGTTCGAATACACCATTATG   477
                    ************************************************************

WT_phop             GAAACGCTTATCCGTAACAACGGTAAAGTGGTCAGCAAAGATTCGCTGATGCTTCAGCTG   720
hns_mutant_phop     GAAACGCTTATCCGTAACAACGGTAAAGTGGTCAGCAAAGATTCGCTGATGCTTCAGCTG   701
14028S_genome_phop  GAAACGCTTATCCGTAACAACGGTAAAGTGGTCAGCAAAGATTCGCTGATGCTTCAGCTG   537
                    ************************************************************

WT_phop             TATCCGGATGCGGAACTGCGGGAAAGTCATACCATTGATGTTCTCATGGGGCGTCTGCGG   780
hns_mutant_phop     TATCCGGATGCGGAACTGCGGGAAAGTCATACCATTGATGTTCTCATGGGGCGTCTGCGG   761
14028S_genome_phop  TATCCGGATGCGGAACTGCGGGAAAGTCATACCATTGATGTTCTCATGGGGCGTCTGCGG   597
                    ************************************************************

WT_phop             AAAAAAATACAGGCCCAGTATCCGCACGATGTCATTACCACCGTACGCGGACAAGGATAT   840
hns_mutant_phop     AAAAAAATACAGGCCCAGTATCCGCACGATGTCATTACCACCGTACGCGGACAAGGATAT   821
14028S_genome_phop  AAAAAAATACAGGCCCAGTATCCGCACGATGTCATTACCACCGTACGCGGACAAGGATAT   657
                    ************************************************************

WT_phop             CTTTTTGAATTGCGCTAATGAATAAATTTGCTCGCCATTTTCTGCCGCTGTCGCTGCGGG   900
hns_mutant_phop     CTTTTTGAATTGCGCTAATGAATAAATTTGCTCGCCATTTTCTGCCGCTGTCGCTGCGGG   881
14028S_genome_phop  CTTTTTGAATTGCGCTAA------------------------------------------   675
                    ******************

WT_phop             TTCGTTTTTTGCTGGCGACAGCCGGCGTCGTGCTGGTGCTTTCTTTGGCATATGGCATAG   960
hns_mutant_phop     TTCGTTTTTTGCTGGCGACAGCCGGCGTCGTGCTGGTGCTTTCTTTGGCATATGGCATAG   941
14028S_genome_phop  ------------------------------------------------------------   675

WT_phop             TGGCGCTGGTCGGCTATAGCGTAAGTTTTGATAAAACCACCTTTCGTTTGCTGCGCGGCG   1020
hns_mutant_phop     TGGCGCTGGTCGGCTATAGCGTAAGTTTTGATAAAACCACCTTTCGTTTGCTGCGCGGCG   1001
14028S_genome_phop  ------------------------------------------------------------   675

WT_phop             AAAGCAACCTGTTTTATACCCTCGCCAAATGGGAAAATAATAAAATCAGCGTTGAGCTGC   1080
hns_mutant_phop     AAAGCAACCTGTTTTATACCCTCGCCAAATGGGAAAATAATAAAATCAGCGTTGAGCTGC   1061
14028S_genome_phop  ------------------------------------------------------------   675
```

Figure S8. Mutation was not occurred in *phoP* loci in the *hns* mutant strain**.** Sequence alignment of *phoP* gene of the hns mutant, WT strain and *S*. Typhimurium ATCC 14028s strain genome using Clustal Omega (https://www.ebi.ac.uk/Tools/msa/clustalo/). Bases with * are strictly conserved.
